# Anxiolytic-like effect of Suanzaoren–Wuweizi herb-pair and evidence for the involvement of the monoaminergic system in mice based on network pharmacology

**DOI:** 10.1186/s12906-022-03829-1

**Published:** 2023-01-09

**Authors:** Jie Liu, Jin-Li Shi, Jian-You Guo, Yi Chen, Xiao-Jie Ma, Sheng-Nan Wang, Zhi-Quan Zheng, Ming-Xuan Lin, Shuai He

**Affiliations:** 1 Capital Institute of Pediatrics, No. 2 Yabao Road, Chaoyang District, Beijing, China; 2 School of Chinese Materia Medica, Beijing University of Chinese Medicine, Yangguang South Street, Fangshan District, Beijing, 102488 China; 3 Key Laboratory of Mental Health, Institute of Psychology, Chinese Academy of Sciences, 4A Datun Road, Chaoyang District, Beijing, 100101 China

**Keywords:** Suanzaoren-Wuweizi, Anxiolytic-like effect, Mechanism, Monoaminergic system, Network pharmacology

## Abstract

**Background:**

Suanzaoren-Wuweizi herb-pair (SWHP), composed of Zizyphi Spinosi Semen (Suanzaoren in Chinese) and Schisandrae Chinensis Fructus (Wuweizi in Chinese), is a traditional herbal formula that has been extensively used for the treatment of insomnia. The study aimed to explore the targets and signal pathways of Suanzaoren-Wuweizi (S-W) in the treatment of anxiety by network pharmacology, and to verify the pharmacodynamics and key targets of SWHP in mice.

**Methods:**

The Traditional Chinese Medicine Systems Pharmacology Database and Analysis Platform (TCMSP) as well as literature mining were used to obtain the main chemical ingredients of Suanzaoren and Wuweizi. The SwissTargetPrediction platform was used to predict drug-related targets. The GeneCards, TTD, DisGeNET and OMIM databases were used to obtain potential targets for the treatment of anxiety with the chemical components of S-W. Drug-disease intersection genes were selected, and a protein-protein interaction (PPI) network was constructed using STRING. The core targets of S-W in the treatment of anxiety were selected according to the topological parameters, and GO functional enrichment as well as KEGG pathways enrichment analyses were performed for potential targets. The relationship network of the “drug-active ingredient-disease-target-pathway” was constructed through Cytoscape 3.8.0. The pharmacodynamics of SWHP in the treatment of anxiety was evaluated by the elevated plus maze (EPM), the light/dark box test (LDB) and the open field test (OFT). The mechanisms were examined by measuring monoamine neurotransmitters in brain of mice.

**Results:**

The results showed that there were 13 active ingredients for the treatment of anxiety in the network. This includes sanjoinenine, swertisin, daucosterol, schizandrer B, wuweizisu C and gomisin-A. Additionally, there were 148 targets, such as AKT1, TNF, SLC6A4, SLC6A3, EGFR, ESR1, HSP90AA1, CCND1, and DRD2, mainly involved in neuroactive ligand-receptor interactions, the Serotonergic synapse pathway and the cAMP signaling pathway. After 1 week of treatment, SWHP (2 and 3 g/kg) induced a significant increase on the percentage of entries into and time spent on the open arms of the EPM. In the LDB test, SWHP exerted anxiolytic-like effect at 2 g/kg. In the open-field test, SWHP (2 g/kg) increased the number of central entries and time spent in central areas. The levels of brain monoamines (5-HT and DA) and their metabolites (5-HIAA, DOPAC) were decreased after SWHP treatment.

**Conclusions:**

The anti-anxiety effect of SWHP may be mediated by regulating 5-HT, DA and other signaling pathways. These findings demonstrated that SWHP produced an anxiolytic-like effect and the mechanism of action involves the serotonergic and dopaminergic systems, although underlying mechanism remains to be further elucidated.

## Background

Anxiety, an aversive emotional state and commonly occurring mental disorder of the central nervous system, is contributing to an ever-increasing health burden worldwide. It’s comprised of a powerful emotional component associated with fearful thoughts and a physiological response. Kessler et al. [1] have reported that anxiety disorders are the most frequently diagnosed mental disorders in the United States with lifetime prevalence in approximately 30% of the population. Benzodiazepines are commonly used in the psychotherapeutic treatment of depression, anxiety and insomnia disorders, and possess sedative, hypnotic, and anti-anxiety properties [2–4]. Long-term use of benzodiazepines, however, is associated with complications such as withdrawal symptoms, therapeutic dose dependence, relapse anxiety, falls and fractures, as well as impairment in long-term cognitive functioning (which can remain for several months after benzodiazepines have been withdrawn) [3–7]. Therefore, the development of other anxiolytic drugs without such adverse effects is important for the treatment of anxiety disorders.

Chinese therapeutic herbs are traditionally used in combination (combined according to their properties so as to extend their abilities) and always demonstrate better pharmacological effects combined than when used alone [8]. Herb-pairs (Yaodui in Chinese), which are unique combinations of two traditional Chinese medicine (TCM) herbs [9], are frequently used as basic composition units in Chinese herbal formulas. They aim to achieve mutual reinforcement, assistance, restraint, and detoxication [10], and are much simpler to make than other complicated formulations [11].

SWHP is a combination of Zizyphi Spinosae Semen (Rhamnaceae, the dried seeds of Ziziphus jujube Mill. var. spinosa (Bunge) Hu ex H.F.Chou, officially recognized in the Chinese Pharmacopoeia) and Schisandrae Chinensis Fructus (Magnoliaceae, the mature fruits of Schisandra chinensis (Turcz.) Baill., officially recognized in the Chinese Pharmacopoeia). Ziziphi Spinosae Semen and Schisandrae Chinensis Fructus are documented in the Divine Husbandman’s Classic of the Materia Medica (the earliest dictionary of Chinese Materia Medica). The former is widely applied in China, Japan, Korea and other oriental countries to treat insomnia and anxiety symptoms [12–16], while the lignan component of the latter also has significant anti-anxiety effects in its own right [17].

SWHP was originally recorded in Puji Fang (Prescriptions for Universal Relief) in the Ming Dynasty, a famous ancient medical manuscript, and was used to treat insomnia and to calm nerves. In TCM, SWHP is used to treat mental diseases in more than 262 prescriptions according to the Dictionary of Chinese Medicine Prescription (including all of the prescriptions of China from the Qin and Han dynasties to the modern times), such as Anshen Pill, Yangxin Anshen Pill and Suanzaoren Powder. They are also applied in Zaoren Anshen Capsules and Jiannao Capsules, to treat the patients suffering from insomnia, forgetfulness, upset, dizziness and neurasthenia [18]. Moreover, it was reported that the combination Suanzaoren with Wuweizi acts synergistically and is beneficial for the treatment of insomnia in rats, where the mechanisms were related to regulating the neurotransmitter of monoamine [19–21]. Our previous study showed that SWHP had an antianxiety effect in RS rat models, and that the treatment may have targeted the mechanism of the ECS-BDNF-ERK signaling pathway [22]. However, the target and signal pathway of SWHP were not predicted systematically and verified. Hence this study was conducted to fill this gap in knowledge and understanding.

Network pharmacology is a new discipline which reveals the regulatory network of drugs in the body from the system level. The mechanism of drug action can be predicted by constructing the complex network relationship among “drugs, active components, targets and diseases”, particularly useful for the mechanism prediction of multi-target drugs developed from Traditional Chinese medicine [23–25].

The monoaminergic system [serotonin (5-HT) and dopamine (DA)] in the brain has been postulated to play an important role in the pathophysiology of anxiety disorders [26–28]. Several preclinical and clinical reports provide evidence to support that a dysfunction of the monoaminergic system may be implicated in the pathophysiology of anxiety disorders [26–30]. Zhang et al. [26] have shown that the modulation of the monoaminergic system forms the basis for the action of anxiolytic drugs, and this hypothesis provides a framework in which the pathophysiology and pharmacotherapy for anxiety may congregate on the modulation of monoaminergic system.

**Fig. 1:**
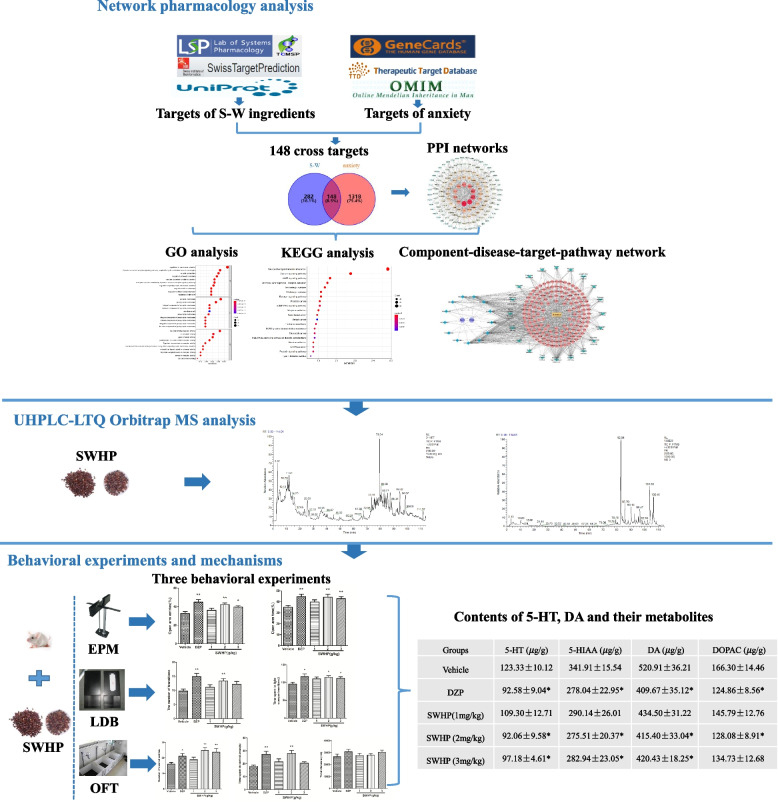
Workflow of this study

The aim of this study is to explore the targets and signal pathways of Suanzaoren-Wuweizi in the treatment of anxiety by utilizing network pharmacology. As well, to verify the anxiolytic-like effect of SWHP by using the elevated plus maze (EPM), the light/dark box test (LDB) and the open field test (OFT). In order to explore the potential mechanisms on monoaminergic system, also examined was the level of monoamines serotonin (5-HT), dopamine (DA) and their metabolites 5-HIAA and DOPAC in brain of mice. The study of these issues not only offers better guidance for the clinical application of SWHP, but also provides foundations for new drug discovery. Figure 1 shows the flow chart of this whole analysis.

## Material and methods

### Data preparation

#### Therapeutic target database searching

The known therapeutic targets of anxiety drugs were obtained from the Genecards (https://www.genecards.org/) [31], Therapeutic Target Database (TTD) (https://db.idrblab.org/ttd/) [32] and OMIM (https://www.omim.org/) [33], with “anxiety” as the search term. By integrating the acquired target protein information and eliminating the repeated targets in the search results, the known targets responsible for the pathogenesis of anxiety were obtained. In the UniProt database (https://www.uniprot.org/) [34], the species was adjusted to “*Homo sapiens*”, while drug targets, protein names, and gene names, were uniformly standardized.

#### Prediction of the component targets for SWHP

The traditional Chinese medicine systems pharmacology database and analysis platform (TCMSP; http://lsp.nwsuaf.edu.cn/tcmsp.php) [35] and Swiss Target Prediction database (http://www.swisstargetprediction.ch/) [36] were used to predict the candidate targets of Suanzaoren and Wuweizi, with the species set as “*Homo sapiens*.” Meanwhile, UniProt database (https://www.uniprot.org) was used to search for protein target IDs and gene IDs, to organize the target information of Suanzaoren and Wuweizi targets, and remove duplicates.

#### Protein-protein interaction (PPI) network analysis

STRING platform (https://string-db.org/) [37] was used to construct an interaction network among the target proteins. The protein type was set as “*Homo sapiens*”, the minimum interaction threshold was adjusted to “Medium confidence”, and other parameters were kept at default values. Cytoscape 3.8.0 software (http://www.cytoscape.org) [38] was used to visualize the PPI network between anxiety-related genes and suanzaoren-wuweizi-target encoding genes.

#### Bioinformatics analysis of SWHP-anxiety targets

To assess the biological significance of specific genes or proteins for Suanzaoren-Wuweizi-anxiety targets, gene ontology (GO) and Kyoto Encyclopedia of Genes and Genomes (KEGG) [39–41] pathway enrichment analyses were performed using DAVID (https://david.ncifcrf.gov/) database. Construct the visualization network diagram of “drug-active ingredient-disease-target-pathway”.

#### Plant materials

The seeds of *Ziziphus jujuba* Mill. var. spinosa (Bunge) Hu ex H.F. Chou were purchased from Sichuan Natural Pharmaceutical co., Ltd. (Sichuan, China). The fruits of Schisandra chinensis (Turcz.) Baill. were purchased from Liaoning Ludan Ltd. (Liaoning, China), and were identified by professor Shi Jin-Li, a botanist at the Beijing University of Chinese Medicine, China. The voucher specimens (No.20160122 of Semen Ziziphi spinosae and No.20150601 of Fructus Schisandrae) were maintained at the Institute of Traditional Chinese Medicine, Beijing University of Chinese Medicine, China. All the materials were dried in the drying room with active ventilation at room temperature (about 22–25 °C) until they achieved constant weight. The plant name was checked against www.theplantlist.org. And the study protocol complies with relevant Chinese institutional, national, and international guidelines and legislation.

#### Reagents and drugs

Diazepam (DZP) was obtained from Yimin Pharmaceutical Factory (Beijing, China, SFDA Approval No. H11020898). 5-HT, 5-hydroxy-3-indoleacetic acid (5-HIAA), dopamine, and 3,4-dihydroxyphenylacetic acid (DOPAC) were purchased from Sigma (St. Louis, MO, USA). Jujuboside A (PubChem CID: 51346169), Jujuboside B (PubChem CID: 24721031), Spinosin (PubChem CID: 155692), Schizandrin (PubChem CID: 23915), Deoxyschizandrin (PubChem CID: 43595) and Schisandrin B (PubChem CID: 158103) were purchased from Sichuan Vicky Biotechnology Co., Ltd. Formic acid, methanol and acetonitrile were purchased from Thermo Fisher Technology Co., Ltd. All the chemicals used in the study were of analytical grade.

#### Preparation of SWHP

SWHP (S:W, 2:1, w/w) were immersed in 80% ethanol (1:8, w/v) and boiled for 1 h. The SWHP was boiled in water (1:10, w/v) again for 1 h. The filtrates from each decoction were mixed and dried under reduced pressure at a temperature of < 60 °C to obtain the powder form of SWHP. The dried powder was then stored at 4 °C before use.

#### Alcohol-water extract analysis by UHPLC-LTQ Orbitrap MS [22]

**Fig. 2:**
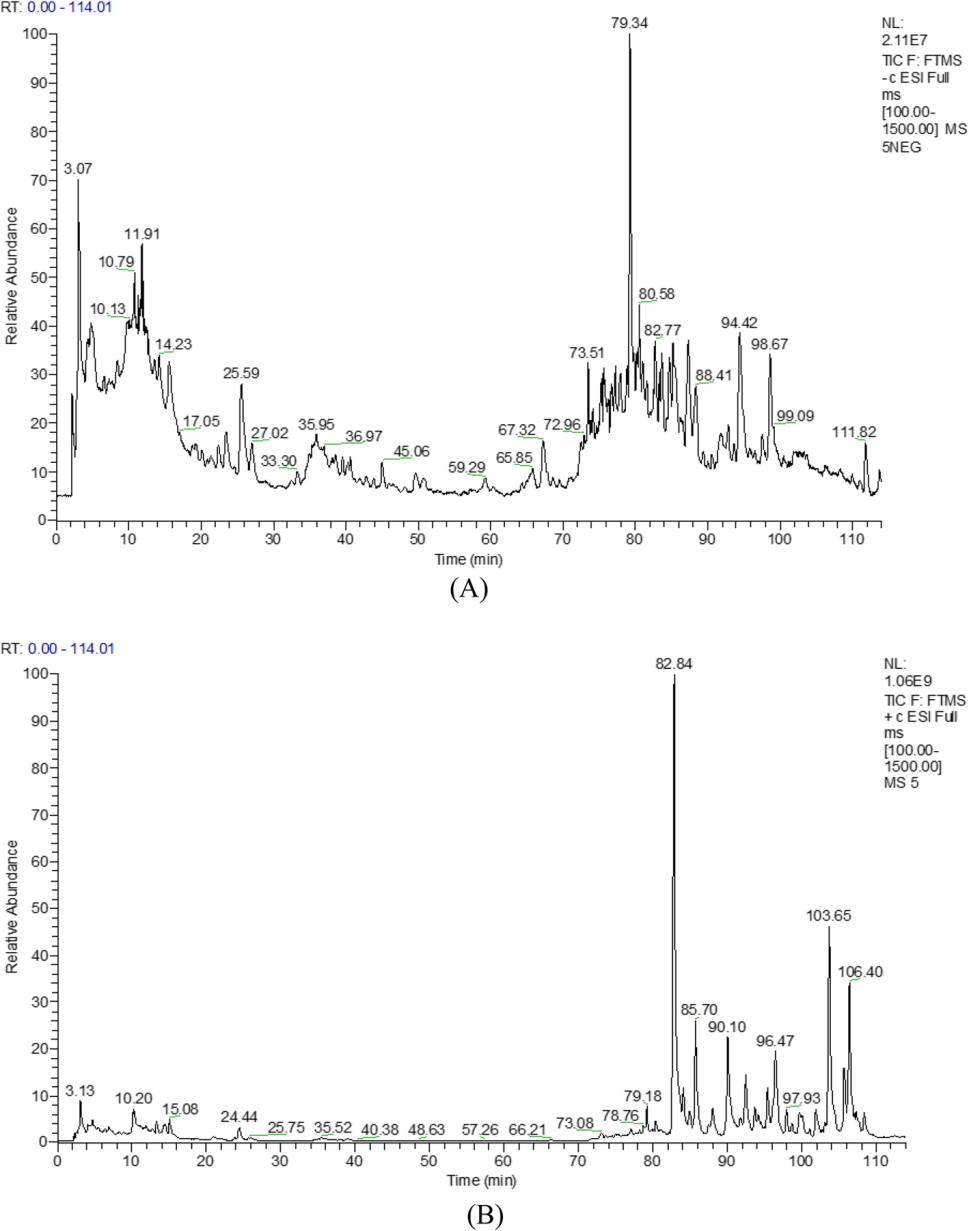
Tic diagram of SWHP in negative ion modes (**A**) and positive ion modes (**B**)

We established the stable conditions of UHPLC-LTQ Orbitrap MS for the analysis of SWHP components in positive and negative ion mode, with good separation and comprehensive cracking information. This part is the chemical composition analysis of SWHP, which was completed and published by our research group. The results showed that a total of 30 compounds were identified including Zizyphusine, Angeloygomisin Q, Schisandrin A, B, C, Gomisin E, G, J, K1, K2, M1, M2, L2, Ceanothic acid, and Lignans (Fig.2**,** Table 1).

**Table 1:** UHPLC-LTQ Orbitrap MS of SWHP

No.	Ion mode	t_R_ (min)	Molecular weight (m/z)	Molecular formula	Fragment ions	Chemical compound
1	Negative	4.59	191.01906	C_6_H_8_O_7_	172,130,128,110,86	Citric acid/isocitrate
2	Negative	9.85	191.019	C_6_H_8_O_7_	172,130,128,110	Citric acid/isocitrate
3	Negative	10.4	205.03429	C_7_H_10_O_7_	173,143,131,111	6-Methyl citrate
4	Negative	14.35	219.04974	C_8_H_12_O_7_	173,157,143,131,111	1,5-Dimethylcitrate
5	Negative	34.93	342.16934	C_20_H_24_NO_4_	297,282,265,237	Zizyphusine
6	Negative	73.59	543.22198	C_29_H_36_O_10_	525,499,481,445	Lancifodilactone C
7	Positive	73.71	501.34177	C_28_H_36_O_8_	455,437	Tigloylgomisin H or aegeloygomisin H
8	Positive	81.64	531.25757	C_29_H_38_O_9_	495,453,425	Angeloygomisin Q
9	Positive	84.94	389.19424	C_22_H_29_O_6_	374,358,342,319	Gomisin J
10	Positive	87.41	515.22632	C_28_H_34_O_9_	469,385,355	Tigloylgomisin P
11	Negative	87.41	401.1593	C_22_H_26_O_7_	354,284,270,257,255,242	3′,4′-Dimethoxybenzoicacid-(3″,4″-dimethoxyphenyl)-methyl-3-oxobutyl ester
12	Positive	87.94	523.22839	C_30_H_34_O_8_	508,493,477,386,315	Benzoylgomisin H
13	Positive	88.36	391.21078	C_22_H_30_O_6_	359,327,289,237,235,205,166	Pregomisin
14	Positive	90.06	523.22894	C_30_H_34_O_8_	493,386,315	Benzoylgomisin H isomer
15	Positive	91.75	387.17938	C_22_H_26_O_6_	372,358,357,356,355,313	Gomisin L2
16	Positive	94.95	403.31021	C_23_H_30_O_6_	388,372,371,340,333,302,301	Schisanhenol
17	Positive	95.41	403.21021	C_23_H_30_O_6_	388,372,371,356,340,333,301	Gomisin K1
18	Negative	96.74	537.20831	C_30_H_34_O_9_	415,385,371	Gomisin G
19	Positive	97.99	403.21021	C_23_H_30_O_6_	388,372,371,356,340,333,301	Gomisin K2
20	Positive	98.66	515.22552	C_28_H_34_O_9_	469,385,355,343,323	Schisantherin B or schisantherin C
21	Positive	99.73	515.22595	C_28_H_34_O_9_	385,355,316	Gomisin E
22	Positive	100.21	387.17983	C_22_H_26_O_6_	355,325,317	Gomisin M1
23	Positive	101.04	387.17896	C_22_H_26_O_6_	355,325,317	Gomisin M2
24	Negative	102.99	485.32553	C_30_H_45_O_5_	439,423	Ceanothic acid
25	Positive	103.65	417.22552	C_24_H_32_O_6_	402,386,370,347,316	Schisandrin A
26	Positive	105.69	401.19507	C_23_H_28_O_6_	386,371,370,331,300	Schisandrin B
27	Positive	106.25	33.117	C_20_H_26_O_4_	300,299,286	Meso-dihydroguaiaretic acid
28	Positive	106.4	401.19485	C_23_H_28_O_6_	386,371,370,331,300	Schisandrin B
29	Positive	107.65	385.16333	C_22_H_24_O_6_	370,355,315,284	Schisandrin C
30	Negative	113.6	279.2319	C_18_H_32_O_2_	261,259,243,83	9,12-Linoleic acid

### Animals

Male ICR mice (18–22 g) were purchased from the Chinese Academy of Military Medical Sciences and kept in cages (25 × 15 × 14 cm) at 22 ± 1 °C on a 12 h/12 h light/dark cycle (light on, 8:00 AM-8:00 PM). The mice were housed six per cage, and water and food were available ad libitum. All of the experiments were performed in a quiet room under dim red light between 8:00 AM and 12:00 PM. All efforts were made to minimize the number of animals used and their suffering. The experimental procedures were approved by the Animal Care and Use Committee of the Institute of Psychology of the Chinese Academy of Sciences (Protocol No. 20170327) and in accordance with the Guide for Care and Use of Laboratory Animals by National Institutes of Health (NIH Pub. No. 85–23, revised 1996).

### Drug administration

**Fig. 3:**
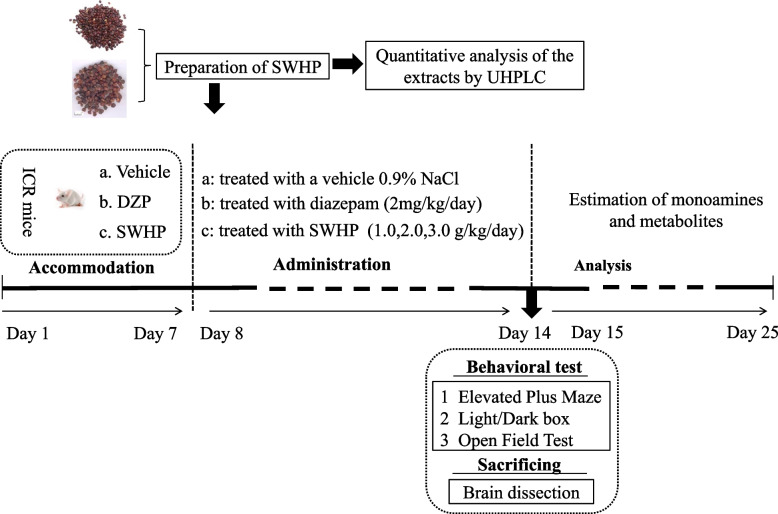
Experimental groups and procedure

The dry powdered extract of SWHP was dissolved in saline (0.05 g/mL). To evaluate the anxiolytic effect of SWHP, the mice were orally administered SWHP (1, 2, and 3 g/kg, the dosage was determined by previous studies [21]) 60 min prior to behavioral testing, or diazepam (2 mg/kg) 30 min before behavioral testing. Diazepam at a dose of 2 mg/kg was chosen as a positive control drug. The dose and administration route for diazepam were based on previous studies [42–44], which was sufficient to induce an anxiolytic effect. All of the drugs were prepared immediately before use and administered orally in a volume of 0.5 mL/25 g body weight for 7 days. All of the behavioral tests were performed on the 7th day of treatment (Fig. 3).

### Behavioral test

#### Elevated plus maze (EPM)

Anxiolytic activity was measured using the EPM [45]. The maze was composed of two opposite open arms (30 cm × 5 cm × 0.2 cm) and two opposite closed arms (30 cm × 5 cm × 15 cm) in a cross configuration [46]. The arms extended from a central platform (5 cm × 5 cm), and the maze was elevated 45 cm above the floor. The entire maze was made of clear Plexiglas. Four 25-W red fluorescent lights were arranged as a cross 100 cm above the maze and provided 200 lx illumination. A video camera was suspended above the maze to record the movements of the mice [47]. The mice (*n* = 12 per group) [47–50] were randomly assigned to five experimental groups: vehicle control, 2 mg/kg diazepam, 1 g/kg SWHP, 2 g/kg SWHP, and 3 g/kg SWHP. The mice were individually placed in the center of the maze facing an open arm, and the number of entries into and the time spent on the closed and open arms were recorded during a 5 min observation period. Arm entries were defined as the placement of all four paws into an arm. The percentage of open-arm entries ([open arm entries/total arm entries] × 100) and the percentage of time spent on the open arms ([time spent on the open arms / total time spent on open arms and closed arms] × 100) were calculated for each animal. If a mouse fell from the apparatus, then it was removed from the study. SWHP and vehicle groups were orally administered 60 min before testing, and the positive control animals were treated with diazepam 30 min before evaluation in the maze. After each trial, the apparatus was cleaned with 70% alcohol.

#### Light/dark box test (LDB)

The LDB test was performed immediately after the EPM test. When the EPM test was completed, the mouse was immediately placed in the light/dark box. The apparatus (45 cm × 21 cm × 21 cm) consisted of two compartments, with one-third painted white and two-thirds painted black. These compartments were separated by a divider with a 3.5 cm × 3.5 cm opening at floor level [51, 52]. The white compartment was illuminated by two 60 W (300 lx) bulbs placed 30 cm above the box. Each mouse was gently placed in the corner of the white compartment away from the dark compartment and monitored for 5 min. The number of transfers from one compartment to the other and time spent in the white compartment were recorded. All of the sessions were recorded by a camera that was linked to a monitor in an adjacent room to avoid distractions [47]. The apparatus was thoroughly cleaned with 70% alcohol after each trial.

#### Open field test (OFT)

The apparatus of the open field was composed of a square arena (60 cm × 60 cm), with a white floor that was divided into 36 squares (10 cm × 10 cm), enclosed by 25-cm-high walls made of black Plexiglas. The arena was illuminated by two 60 W red lamps placed over the center. The lamps were close to each other, placed 120 cm above the floor and provided 100 lx illumination in the testing room [47]. The 16 squares in the center represented an exposed field. The other 20 squares that were adjacent to the walls represented a protected field (safe areas). The test was initiated by placing a single mouse in the middle of the arena and allowing it to move freely for 5 minutes [53]. The number of central entries, the time spent in the central area and the total distances were recorded by an automatic video tracking system. The OFT was performed 60 min after the final SWHP treatment and 30 min after diazepam treatment. After each trial, the apparatus was wiped clean with 70% alcohol to remove any traces left behind by previous animals.

#### Determination of monoamines and metabolites

The mice were decapitated by cervical dislocation immediately after the OFT. The brains were dissected and immediately placed on ice. The tissue samples were weighed and stored at − 80 °C until homogenization. The brain tissue was manually homogenized with three volumes (w/v) of ice-cold 0.1 M perchloric acid (100 𝜇L/mg wet weight) that contained 0.1 mM ethylenediaminetetra-acetic acid (EDTA). After homogenization and centrifugation at 12,000×g at 4 °C for 10 min, 20 𝜇L of the tissue homogenate supernatant was injected directly into a high-performance liquid chromatography (HPLC) system that was equipped with an electrochemical detector (Waters ECD 2465, Milford, Massachusetts, USA). The mixed standard was used as a reference. The levels of monoamines (5-HT, DA, 5- HIAA, and DOPAC) in the samples were expressed as nanograms per gram of fresh weight of tissue [54]. The HPLC system used a reversed-phase C18 column (2.1 mm × 150 mm, 3 𝜇m, Waters Atlantis). The mobile phase consisted of 50 mM citric acid-sodium citrate (pH 3.5), 0.3 mM Na2-EDTA, 1.8 mM dibutylamine, and 4% methanol. The flow rate was 0.35 mL/min, and the detector potential was + 0.75 V.

#### Statistical analysis

All data obtained is presented as mean ± standard error of the mean (SEM). First, check whether the data conforms to the normal distribution. When the data accorded with normal distribution, the statistical analysis was performed using one-way analysis of variance (ANOVA) followed by the Student–Newman–Keuls post hoc test and GraphPad Prism 5.0 software. When the data did not conform to the distribution of positive and negative, a nonparametric test was used to analyze the data. In cases of significant variation, the individual values were compared using Dunnett’s test. Values of *p* < 0.05 were considered statistically significant.

## Results

### Identification of anxiety-related targets

From Genecards, TTD, DisGeNET and OMIM platforms, a total of 1425, 57, 1048 and 52 anxiety-related targets were obtained respectively. After merging the targets predicted by the four platforms and deleting duplicates, a total of 1318 anxiety-related targets were identified.

### Identification of S-W targets

From TCSMP and Swiss Target Prediction databases, a total of 423 and 449 targets (respectively) of Suanzaoren and Wuweizi were retrieved. After merging the targets predicted by the two databases and removing duplicates, a total of 282 targets were identified.

### PPI network of anti-anxiety targets for S-W

**Fig. 4:**
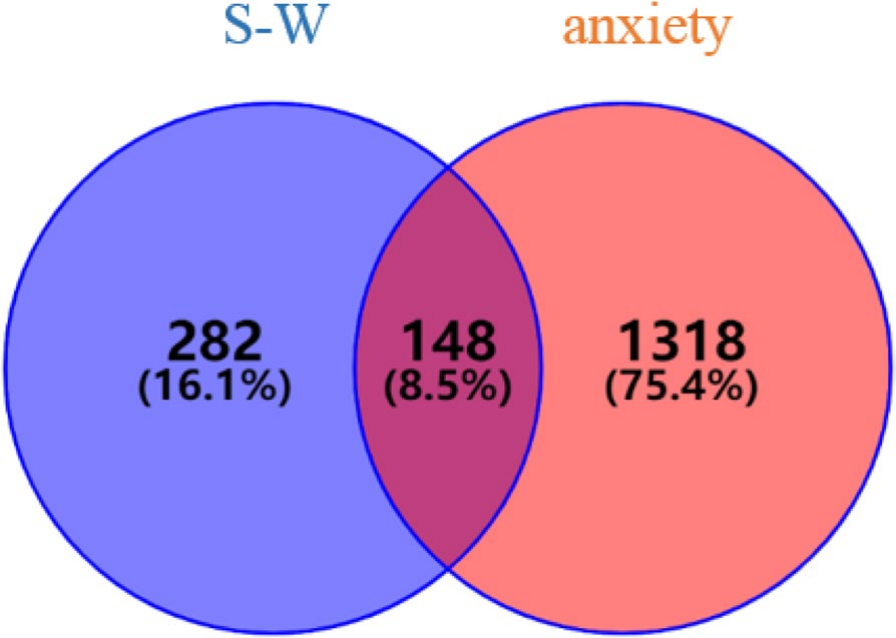
Venny diagram of S-W prediction targets and anxiety targets

**Fig. 5:**
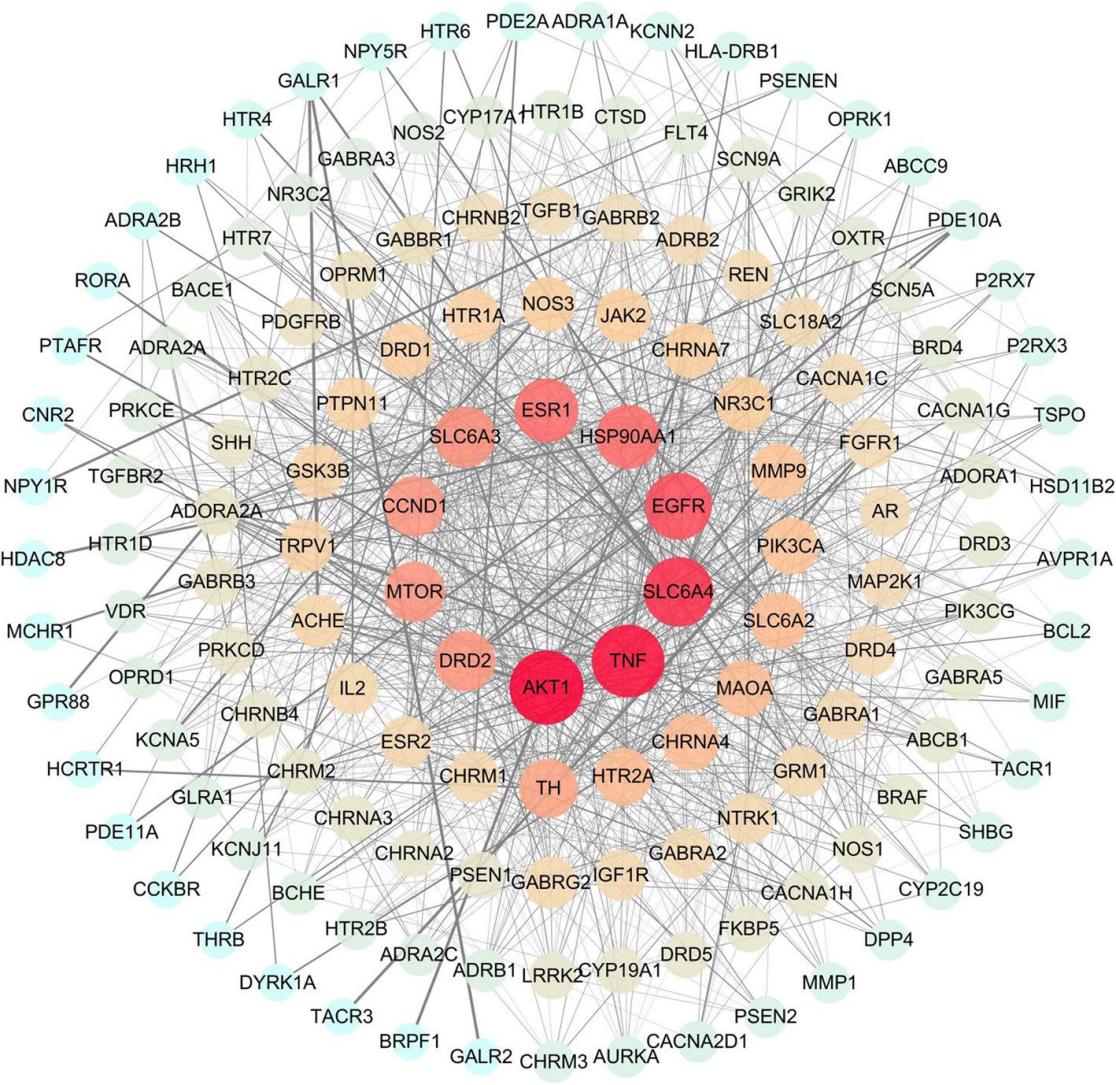
PPI network map of common targets of S-W and anxiety

The targets of S-W and anxiety were screened, and 148 common targets were found between S-W and anxiety (Fig. 4). All interaction targets were imported into the STRING platform for PPI network analysis (Fig. 5). There were 148 nodes and 1068 edges in the network, and the average degree value was 14.4. A network analyzer tool was used for topology analysis, and genes with scores greater than average were selected as key targets by degree sorting. A total of 66 key targets were screened out. As can be seen from the figure, AKT Serine/Threonine Kinase 1 (AKT1), Tumor Necrosis Factor (TNF), Solute Carrier Family 6 Member 4 and 3 (SLC6A4, SLC6A3), epidermal growth factor receptor (EGFR), Estrogen receptor 1 (ESR1), Heat Shock Protein 90 Alpha Family Class A Member 1 (HSP90AA1), Cyclin D1 (CCND1), Dopamine Receptor D2 (DRD2), and Mechanistic Target of Rapamycin Kinase (MTOR) may be the key targets of S-W in the treatment of anxiety disorders.

### GO and KEGG pathway enrichment by S-W for potential anxiety targets

**Fig. 6:**
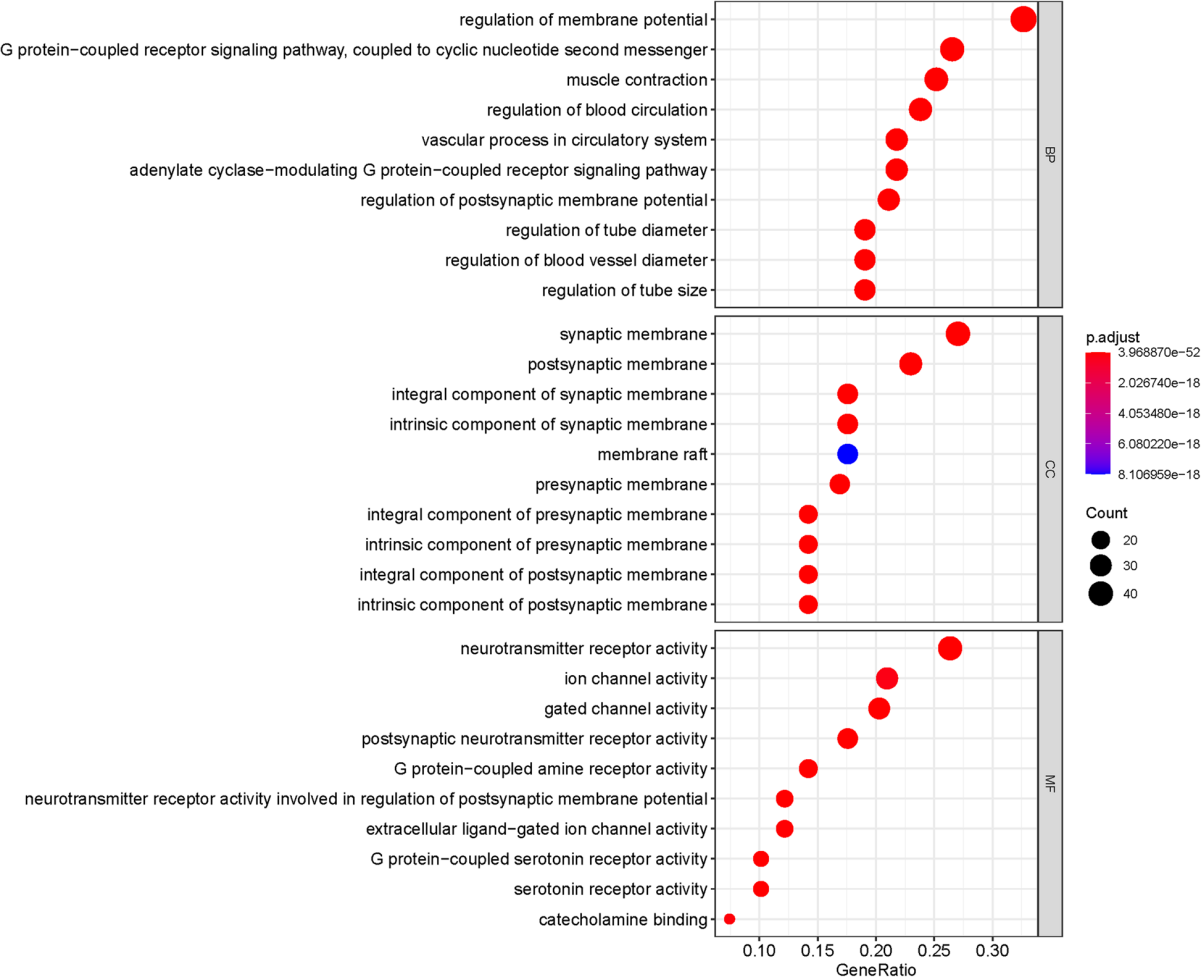
GO enrichment of related target genes of S-W and anxiety

GO enrichment can be found in 1700 biological processes, 111 items related to cell composition and 162 items related to molecular function. The key targets were mainly involved in the biological process (BP) of the membrane potential regulation, G protein coupled receptor signaling pathway, blood circulation regulation, cellular component (CC) of presynaptic membrane, postsynaptic module, and has molecular function (MF) of processing neurotransmitter receptor activity, G protein-coupled receptor activity and ligand-gated ion channel activity (Fig. 6).

**Fig. 7:**
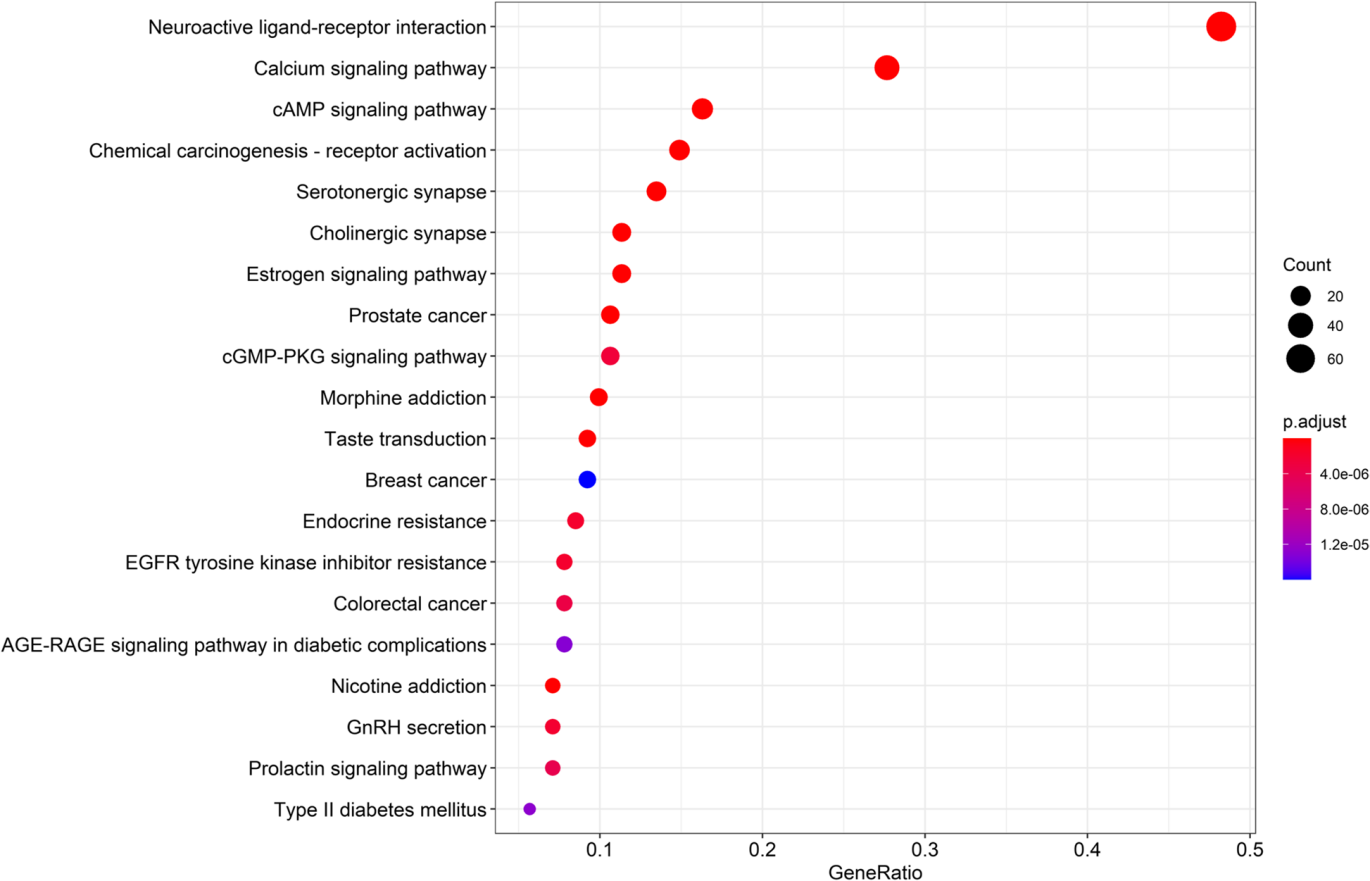
KEGG pathways enrichment analysis of related target genes of S-W and anxiety

KEGG pathway analysis revealed that there were eleven pathways associated with the anti-anxiety effects of S-W. These include: the neuroactive ligand-receptor interaction pathway, Serotonergic synapse, Cholinergic synapse, cAMP signaling pathway, cGMP-PKG signaling pathway, Calcium signaling pathway, Estrogen signaling pathway, GnRH secretion, Prolactin signaling pathway, Endocrine resistance and EGFR tyrosine kinase inhibitor resistance (Fig. 7).

### Construction of “drug-active component-disease-target-pathway” network

**Fig. 8:**
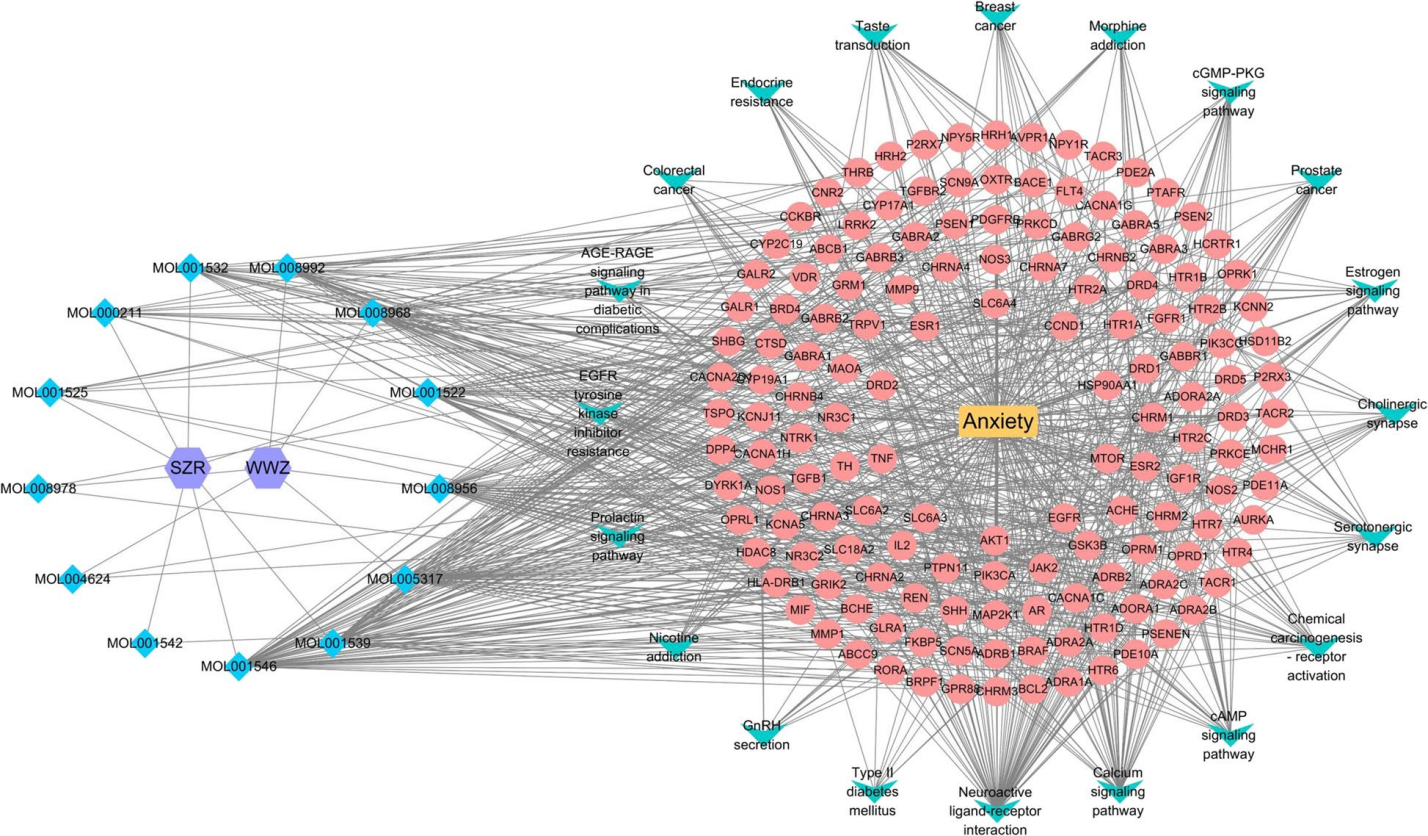
Interaction Network of Drug-active Component-disease-target-pathway

Cytoscape 3.8.0 software was used to construct the visualization network diagram of “drug-active ingredient-disease-target-pathway”, as shown in Fig. 8. The network contains 13 chemical components, 148 action targets and 20 important pathways. Each chemical component in S-W corresponds to multiple targets and pathways, which further proves that S-W can treat anxiety disorders through multi-component and multi-target synergistic action.

### Effects of SWHP in the EPM, LDB and OFT

**Fig. 9:**
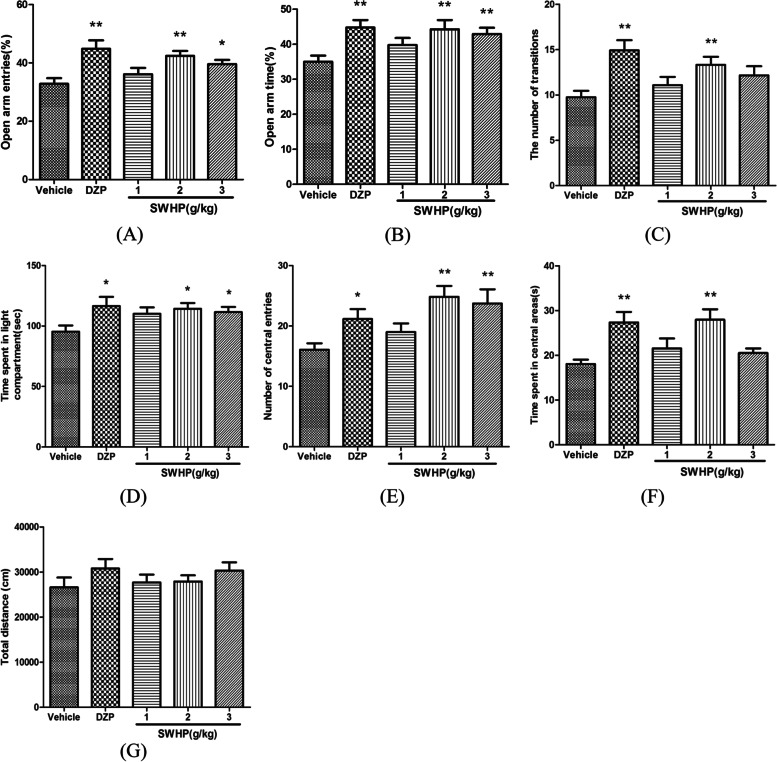
Effect of SWHP on the open arm entries (**A**) and the percentage of time spent in open arms (**B**) in the elevated plus maze, effect on the number of transitions (**C**) and the time spent in light compartment (**D**) in the light/dark box test, and effect on the number of center entries (**E**), time spent in central areas (**F**) and the total distances (**G**) in the open field test in mice

We examined the effects of SWHP in the EPM in comparison with the vehicle group. As shown in Fig. 9A and B (Supplementary Materials), there were significant differences among the five groups in both the percentage of open-arm entries [F (4,55) = 5.275, *P* < 0.01] and the percentage of time spent on the open arms [F (4,55) = 3.747, *P* < 0.01]. After one-week of treatment, SWHP (2 and 3 g/kg) led to a significant increase in the number of entries into the open arms from the vehicle. From 32.83 ± 1.93% to 42.42 ± 1.68% and 39.58 ± 1.45% respectively for 2 and 3 g/kg treatments (*n* = 12, *P* < 0.01, *P* < 0.05; Fig. 9A) and the time spent in the open arms from the vehicle from 35.00 ± 1.75% to 44.25 ± 2.63% and 42.92 ± 1.75% respectively for 2 and 3 g/kg treatments (*n* = 12, *P* < 0.01, *P* < 0.01; Fig. 9B). SWHP at the lower dose (1 g/kg) did not generate significant effect on the percentage of open-arm entries or the percentage of time spent on the open arms. Meanwhile, DZP induced a significant increase in the percentage of open-arm entries from 32.83 ± 1.93% to 44.83 ± 2.90% (*n* = 12, *P* < 0.01; Fig. 9A) and the percentage of time spent on the open arms to 44.75 ± 2.15% (*n* = 12, *P* < 0.01; Fig. 9B) when compared with the vehicle group.

Figure 9C and D showd the effects of SWHP on the number of transitions and time spent in the light compartment in the LDB test in mice. Marked increases were observed in both the number of transitions [F (4,55) = 4.475, *P* < 0.01] and time spent in light compartment [F (4,55) = 2.526, *P* < 0.05]. After one-week of treatment, SWHP at the dose of 2 g/kg significantly increased the number of transitions from the vehicle from 9.75 ± 0.72 to 13.33 ± 0.88 (*n* = 12, *P* < 0.01; Fig. 9C) and time spent in light compartment from the vehicle from 95.35 ± 5.10 to 114.30 ± 4.72 (*n* = 12, *P* < 0.05; Fig. 9D). SWHP at the dose of 3 g/kg significantly increase the time spent in light compartment to 111.54 ± 4.21 (*n* = 12, *P* < 0.05; Fig. 9D). DZP also induced effects of increasing the number of transitions (14.92 ± 1.14, *n* = 12, *P* < 0.01; Fig. 9C) and time spent in light compartment (116.43 ± 7.66, *n* = 12, *P* < 0.05; Fig. 9D) when compared with the vehicle group.

The results for the OFT are shown in Fig. 9E-G. There were significant differences in the number of center entries [F (4,55) = 4.308, *P* < 0.01] and the time spent in central areas [F (4,55) = 5.339, 𝑃 < 0.01], but not in the total distances [F (4,55) = 0.941, *P* = 0.447; Fig. 9G]. Compared with the vehicle group, SWHP (2 g/kg) and DZP significantly increased the number of central entries from 16.08 ± 1.06 to 24.83 ± 1.82 (*n* = 12, *P* < 0.01; Fig. 9E) and 21.17 ± 1.65 (*n* = 12, *P* < 0.05; Fig. 9E) respectively as well, the time spent in central areas increased from 18.06 ± 0.98 to 27.96 ± 2.34 (*n* = 12, *P* < 0.01; Fig. 9F) and 27.35 ± 2.34 (*n* = 12, *P* < 0.01; Fig. 9F) for SWHP (2 g/kg) and DZP respectively. In addition, SWHP (3 g/kg) also significantly increased the number of central entries from 16.08 ± 1.06 to 23.75 ± 2.33 (*n* = 12, *P* < 0.01; Fig. 9E).

Mice were administered vehicle, SWHP (1,2,3 g/kg) or DZP for 7 days. Values were presented as mean ± S.E.M. (*n* = 12). * *P* < 0.05 and ** *P* < 0.01 as compared with the vehicle group. One-way ANOVA with Student-Newman-Keuls post hoc test.

### Effects of SWHP on monoamine neurotransmitters and their metabolites

Table 2 illustrates the effects of SWHP on monoamine neurotransmitters and their metabolites. There were significant differences on the content of 5-HT, DA [F (4,54) = 2.94, *P* < 0.05, F (4,54) = 2.27, *P* < 0.05] and their metabolites 5-HIAA and DOPAC [F (4,54) = 2.60, *P* < 0.05, F (4,54) = 2.51, *P* < 0.05]. Compared with the vehicle group, SWHP (2 and 3 g/kg) and DZP significantly decreased the content of 5-HT from 123.33 ± 10.12 to 92.06 ± 9.58, 97.18 ± 4.61 and 92.58 ± 9.04 (*n* = 11–12, *P* < 0.05) respectively. As well, decreased the content of 5-HIAA from 341.91 ± 15.54 to 275.51 ± 20.37, 282.94 ± 23.05 and 278.04 ± 22.95 (*n* = 11–12, *P* < 0.05), and decreased the content of DA from 520.91 ± 36.21 to 415.40 ± 33.04, 420.43 ± 18.25 and 409.67 ± 35.12 (*n* = 11–12, *P* < 0.05). SWHP (2 g/kg) and DZP also significantly decreased the content of DOPAC from 166.30 ± 14.46 to 128.08 ± 8.91 and 124.86 ± 8.56 (*n* = 11–12, *P* < 0.05) when compared with the vehicle group (Supplementary materials are available online).

**Table 2:** Effects of SWHP on Monoamine Neurotransmitters and their Metabolites

Groups	5-HT (𝜇g/g)	5-HIAA (𝜇g/g)	DA (𝜇g/g)	DOPAC (𝜇g/g)
Vehicle	123.33 ± 10.12	341.91 ± 15.54	520.91 ± 36.21	166.30 ± 14.46
DZP	92.58 ± 9.04*	278.04 ± 22.95*	409.67 ± 35.12*	124.86 ± 8.56*
SWHP(1 mg/kg)	109.30 ± 12.71	290.14 ± 26.01	434.50 ± 31.22	145.79 ± 12.76
SWHP (2 mg/kg)	92.06 ± 9.58*	275.51 ± 20.37*	415.40 ± 33.04*	128.08 ± 8.91*
SWHP (3 mg/kg)	97.18 ± 4.61*	282.94 ± 23.05*	420.43 ± 18.25*	134.73 ± 12.68

Mice were administered vehicle, SWHP (1, 2, 3 g/kg) or DZP for 7 days. Values were presented as mean ± S.E.M. (*n* = 11–12). *P < 0.05 and **P < 0.01 as compared with the vehicle group. One-way ANOVA with Student-Newman-Keuls post hoc test.

## Discussion

Over time medical scientists have come to the realization that the pathogenesis and progression of diseases are often so complicated, that the therapeutic effect of one single drug may be modest, or hampered by various side effects and resistances in clinic [55, 56]. Herb pairs, a centralized representative of Chinese herbal compatibility, are the most fundamental and the simplest form of multi-herb formulae and are of great importance in the studies of herb compatibility considering their simplicity yielding the basic characteristics of complex formulae.

During the study, network pharmacology was used to reveal the synergistic effect of multi-target, multi-component and multi-pathway herb-pair treatments at the molecular level. The components of SWHP were detected in the UHPLC-LTQ Orbitrap MS, including alkaloids (Zizyphusine), flavonoids (Ceanothic acid, Citric acid, Lancifodilactone C) in Ziziphi spinosae Semen, and lignans (Schisandrin A, B, C, Schisantherin B, C, Gomisin E, G, J, K1, K2, M1, M2, L2, Angeloygomisin Q) in Schisandrae chinensis Fructus, which is consistent with the types of chemical components predicted by network pharmacology of SWHP. This is also consistent with the literature, which report the components in Ziziphi spinosae Semen [57–59] and Schisandrae chinensis Fructus [60–62]. Through the prediction of drug targets and disease targets, 282 S-W targets and 1318 anxiety disorder targets were analyzed. The important targets of S-W in the treatment of anxiety disorder were identified. After PPI analysis, 66 key targets were screened out.

The target after the compatibility of S-W mainly focuses on AKT1, TNF, SLC6A4, SLC6A3, and DRD2. AKT/protein kinase B signaling pathway regulates cell growth and proliferation, and participates in cell processes, including apoptosis and glucose metabolism. Activation of AKT requires the activation of PI3K and PI3K-AKT pathway, which play an important role in improving anxiety and depression [63]. Related research shows that TNF- α is related to anxiety disorders, as it can promote the release of adrenocortical hormone through the hypothalamus pituitary adrenal axis system, thus causing neuroendocrine disorder and promoting the occurrence of anxiety and depression [64]. SLC6A4 gene can encode serotonin transporters, and SLC6A3 can encode dopamine transporters. In the stress response, the increased expression of the 5-HT2A receptor will enhance the anxiety and depression-like behavior of animals. When it is down regulated, the symptoms will be significantly reduced [65, 66]. Dopaminergic synapses are involved in the synthesis of dopamine (DA) in the brain. DA plays an important role in the generation and transmission of pleasant feelings and the storage of pleasant information. The activation of corresponding membrane receptors is the key to the role of DA. DA receptors have two subtypes, which can be divided into DRD1 and DRD2. When DRD2 is activated, the level of cAMP in cells decreases, and the expression of DRD2 increases significantly, which not only inhibits the production of cAMP but also affects the production of DA, thus leading to depression and anxiety [67].

Through the construction of network pathways, GO enrichment analysis, and KEGG pathway enrichment analysis, we found that the active components of S-W may play a role in the treatment of anxiety by participating in neuroactive ligand-receptor interaction, serotonin synapse and the cAMP signaling pathway. It is known from literature that the receptor biogenic imines contained in the neuroactive ligand-receptor interaction signal pathway is an essential stimulating nerve tissue molecule, which controls and regulates many important biological functions after binding to the corresponding receptors. For example, emotion, memory and the endocrine system. The disorder of this pathway or the down-regulation of the receptor will subsequently cause anxiety [68, 69]. The serotonin synaptic pathway was should also be considered; serotonin is a well-known molecule that produces a pleasant mood, and can participate in regulating mood, energy and memory. Serotonin is also related to the occurrence of anxiety disorders [70, 71]. The cAMP signaling pathway is involved in producing an anti-anxiety effect through the regulation of cAMP, which reduces the level of intracellular cAMP, resulting in a specific anxiolytic-like effect [72, 73].

In the present study we provided convincing evidence that SWHP extract administered by oral route produces a specific anxiolytic-like effect in EPM, LDB and OFT after one-week treatment in mice and the effect is involved with the monoaminergic system.

In the present study we provide convincing evidence that SWHP extract administered by oral route produces a specific anxiolytic-like effect in the EPM, LDB and OFT after one-week of treatment in mice, and the effect is involved with the monoaminergic system. The EPM is considered to be an etiologically valid animal model of anxiety because it uses natural stimuli (fear of a novel open space and fear of balancing on a relatively narrow, raised platform) that can induce anxiety in humans [74–76]. The time spent on the open arms and the number of entries into the open arms is used to assess a state of fear or anxiety [74, 77]. An anxiolytic agent increases the frequency of entries into the open arms and increases the time spent in open arms of the EPM. In the present study, diazepam (2 mg/kg) significantly increased time spent and number of entries into the open arms of the EPM. Moreover, the anxiolytic effect of SWHP (2 and 3 g/kg) was similar to that of DZP.

The light/dark test is based on the innate aversion rodents have to brightly illuminated areas and on the spontaneous exploratory behavior of rodents in response to mild stressors, that is, novel environment and light [78, 79]. Previous studies reported that this test is sensitive to benzodiazepines. Benzodiazepines may increase the number of visits and/or the time spent in the brightly lit area [80]. Our findings clearly suggested that DZP (2 mg/kg) and SWHP (2 g/kg) significantly increased the number of transitions between the compartments and the time spent in the light compartment of the LDB. SWHP at the dose of 3 g/kg also significantly increased the time spent in the light compartment. These results indicate that SWHP has anxiolytic activity.

The OFT is used in studies of the neurobiological basis of anxiety and screening for anxiolytic compounds [81]. The time spent in the central area and the number of central entries served as indices of anxiety and the distance was considered the index of locomotor activity [82, 83]. Activity in the central part of the open field is thought to be correlated with a degree of fear, while the behavior in the peripheral zone and along the walls of the field is thought to reflect general activity [83, 84]. All the data discussed up to here strongly suggests that the mice treated with SWHP (2 g/kg) and DZP (2 mg/kg) significantly increased the number of central entries and time spent in central areas. And the number of central entries was also significantly increased by SWHP at the dose of 3 g/kg. Therefore, SWHP showed a significant anxiolytic-like effect in this paradigm.

As a brain mechanism mediating effects of environmental factors, we focused on serotonin (5-HT) and DA systems in the present study. The current research on the etiology and pathogenesis of anxiety disorders has demonstrated that 5-HT is one of the key neurotransmitters modulating anxiety [85]. 5-HT and its metabolite (5-HIAA), as measured by HPLC, revealed a significant decrease in the SWHP (2 and 3 g/kg) and DZP (2 mg/kg) treated mice. The central dopaminergic system is considered a crucial factor in anxiety disorders. Foot-shock and anxiogenic drugs markedly increase cortical dopamine output in normal rats, and chronic treatment with imipramine completely inhibits these changes [86]. The present results are consistent with these reports. SWHP and DZP significantly reduced the tissue concentration of DA and DOPAC, the major metabolites of DA, in brain homogenates. Hence, these results further support the hypothesis that the anxiolytic effect of SWHP is mainly mediated via the monoaminergic system, including 5-HTergic and DAergic systems.

Notably, the neurotransmitter system predicted in network pharmacology not only included the 5-HT system and DA system, but also included the GABA system and cAMP signal pathway as predicted key pathways. Therefore, it is necessary to also determine GABA, which was reported to be one of the mechanisms of DZP, and the key proteins in the cAMP signal pathway, such as PKA, ERK, and RAP1a in the later stage. This is required in order to more comprehensively explain the anti-anxiety mechanism of SWHP.

## Conclusions

In summary, the mechanism of SWHP for the treatment of anxiety was analyzed, and the reliability of the network pharmacology prediction was verified by animal experiments. Our findings open new perspectives for understanding SWHP for anxiety, and indicates that SWHP exerts an anxiolytic-like effect in the EPM, LDB and OFT, without affecting locomotor activity, in mice. In addition, this work provides evidence that the anxiolytic effect of SWHP appears to be mediated through the monoamine neurotransmitter levels. The findings of this study provide reference and a scientific basis for further study into the anti-anxiety effect of SWHP, as it provides an alternative safe and effective treatment with less side effects.

## Data Availability

All data and materials are described within the article. The corresponding author will provide if requested.

## Abbreviations

5-HT: 5-hydroxytryptamine
5-HIAA: 5-hydroxy-3-indoleacetic acid
AKT1: AKT Serine/Threonine Kinase 1
ANOVA: Analysis of variance
CCND1: Cyclin D1
DA: Dopamine
DOPAC: 3,4-dihydroxyphenylacetic acid
DRD2: Dopamine Receptor D2
DZP: Diazepam
EDTA: Ethylenediaminetetra-acetic acid
EGFR: Epidermal growth factor receptor
EPM: Elevated plus maze
ERK: Extracellular regulated protein kinases
ESR1: Estrogen receptor 1
GABA: γ-aminobutyric acid
GO: Gene ontology
HSP90AA1: Heat Shock Protein 90 Alpha Family Class A Member 1
KEGG: Kyoto Encyclopedia of Genes and Genomes
LDB: Light/dark box
MTOR: Mechanistic Target of Rapamycin Kinase
OFT: Open field test
PKA: Protein Kinase A
PPI: Protein-protein interaction
RAP1a: Ras-associated protein 1a
SEM: Standard error of the mean
SLC6A3: Solute Carrier Family 6 Member 3
SLC6A4: Solute Carrier Family 6 Member 4
SWHP: Suanzaoren-Wuweizi herb-pair
TCMSP: Traditional Chinese Medicine Systems Pharmacology Database and Analysis Platform
TNF: Tumor Necrosis Factor
